# Calcium ions released from alginate hydrogel promote wound healing by enhancing fibroblast activity

**DOI:** 10.3389/fbioe.2026.1828848

**Published:** 2026-06-19

**Authors:** Lei Zhang, Ying Luo, Shubo Liu, Huiting Deng, Yingtang Gao

**Affiliations:** 1 School of Medicine, Nankai University, Tianjin, China; 2 Department of Clinical Laboratory, Gansu Provincial Hospital, Lanzhou, China; 3 Tianjin Key Laboratory of Extracorporeal Life Support for Critical Diseases, Tianjin Institute of Hepatobiliary Disease, Nankai University Affiliated Third Center Hospital, Tianjin, China; 4 Tianjin Key Laboratory of Extracorporeal Life Support for Critical Diseases, Tianjin Institute of Hepatobiliary Disease, Central Hospital, Tianjin University/Tianjin Third Central Hospital, Tianjin, China; 5 The Third Central Clinical College of Tianjin Medical University, Tianjin, China; 6 Artificial Cell Engineering Technology Research Center, Tianjin, China

**Keywords:** alginate hydrogel, calcium crosslinking, diabetic wound, fibroblast activation, wound healing

## Abstract

Hydrogels are widely used as wound dressings owing to their biocompatibility, high water content, and ability to mimic extracellular matrix functions. Alginate (ALG) is a natural polysaccharide that forms ionically crosslinked hydrogels in the presence of calcium, where Ca^2+^ not only stabilizes the crosslinked network but also regulates cell behavior, which is essential for wound repair. In this study, ALG hydrogels were prepared with CaCl_2_ at 20, 50, 100, and 200 mM, and their gelation, rheological properties, calcium ion release, and cellular responses were systematically evaluated. The 100 mM CaCl_2_ formulation exhibited the optimal mechanical stability and bioactivity, markedly promoting fibroblast proliferation, migration, and extracellular matrix organization *in vitro*. Application of this hydrogel to a rat model with full-thickness skin defects significantly accelerated wound closure and tissue regeneration. Transcriptomic analysis further confirmed activation of the calcium signaling pathway in fibroblasts. These findings highlight the pivotal role of Ca^2+^ in orchestrating fibroblast activity, providing an optimized alginate-based dressing for effective wound healing.

## 1 Introduction

Wound healing is a dynamic and tightly regulated complex process that encompasses the hemostasis, inflammation, proliferation, and remodeling phases (Freedman et al., 2023; Zhang et al., 2024). Fibroblasts are indispensable regulators of wound healing across these phases. They orchestrate extracellular matrix deposition, modulate inflammatory signaling, and drive tissue repair and scar formation (Zhang et al., 2024; Li et al., 2024; Gajbhiye and Wairkar, 2022). Despite significant advancements in clinical wound care, challenges such as delayed healing, infection, and impaired tissue regeneration remain substantial, particularly in the context of chronic wounds. Owing to their hydrophilic polymer networks, hydrogels exhibit excellent biocompatibility, biodegradability, antimicrobial properties, and hemostatic capability, making them highly suitable as wound dressing materials (Zhao et al., 2020; Firlar et al., 2022; Farazin et al., 2023; Luo et al., 2025). Notably, alginate (ALG) hydrogels, derived from natural polysaccharides, are widely utilized in wound healing applications because of their capability to rapidly form gels in the presence of divalent cations (Chelu et al., 2024; Man et al., 2022).

Alginate polymers exhibit high affinity toward divalent cations such as Mg^2+^, Ca^2+^, Sr^2+^, and Ba^2+^, which can interact with guluronic acid (G) blocks in the proposed “egg-box” model to form crosslinked networks (Rajalekshmy et al., 2024; Wang et al., 2023). Among these cations, calcium ions (Ca^2+^) play a pivotal role in stabilizing the hydrogel structure through specific junctions (Morris et al., 1978; Kumar et al., 2023; Wang et al., 2024). Notably, the Ca^2+^ concentration considerably affects the crosslinking density and mechanical properties of alginate hydrogels, thereby influencing their functional performance in biomedical applications (Li et al., 2022). Ca^2+^-crosslinked alginate (ALG/CaCl_2_) hydrogels have been extensively explored for various therapeutic applications, including wound healing (Li et al., 2022; Zhang et al., 2021) and bone tissue regeneration (Wei et al., 2023; Chen and Lv, 2022).

Furthermore, Ca^2+^ serves as one of the most prevalent second messengers in cellular signaling, orchestrating a wide range of cellular processes, such as transcription, apoptosis, adhesion, activation, exocytosis, metabolism, and proliferation (Navarro-Requena et al., 2018; Jeong et al., 2018). In fibroblasts, Ca^2+^ signaling influences migration and extracellular matrix deposition, both of which are essential for wound healing (Kumar et al., 2023; Navarro-Requena et al., 2018; Schreiber, 2005; Doyle et al., 1996; Lansdown, 2002; Subramaniam et al., 2021). These observations suggest that Ca^2+^ not only affects the physicochemical properties of alginate hydrogels but may also play a direct role in their biological and therapeutic functions. Previous studies have demonstrated that Ca^2+^ released from ALG/CaCl_2_ hydrogels can modulate fibroblast behavior, including proliferation, viability, and migration (Tordi et al., 2025). However, the influence of varying Ca^2+^ concentrations on the mechanical and biological properties of these hydrogels has not been comprehensively evaluated, particularly in terms of balancing mechanical stability with fibroblast-mediated bioactivity relevant to wound healing. Therefore, this study examines the effects of varying Ca^2+^ concentrations on the structural, mechanical, and biological properties of ALG/CaCl_2_ hydrogels, with a particular emphasis on their potential applications in wound healing.

In this study, we addressed these gaps by using 2% (w/v) ALG as the base. We prepared hydrogels crosslinked with CaCl_2_ at concentrations of 20, 50, 100 and 200 mM and systematically evaluated their gelation behavior, rheological properties, 24-h calcium ion release, and effects on L929 fibroblast proliferation and migration. The formulation with the most favorable balance, 100 mM CaCl_2_, was then applied to a rat model with full-thickness skin defects. To elucidate the molecular mechanisms responsible for enhanced healing, we conducted transcriptomic analysis on wound tissues treated with this hydrogel.

## 2 Materials and methods

### 2.1 Materials

Sodium alginate (viscosity: 200 mPas) and calcium chloride dihydrate (CaCl_2_.2H_2_O, analytical grade) were purchased from Aladdin Chemical Reagent Co., Ltd (Shanghai, China). Dulbecco’s modified Eagle medium (DMEM), fetal bovine serum (FBS), penicillin–streptomycin, and trypsin–EDTA were purchased from Gibco (Thermo Fisher Scientific, Waltham, MA, United States). Mouse fibroblast cell line L929 was obtained from HyCyte Biotechnology (Suzhou, China). All chemicals and reagents were of analytical grade and were used without further purification.

### 2.2 Preparation of ALG hydrogels with different Ca^2+^ concentrations

ALG hydrogels were prepared through ionic crosslinking using CaCl_2_ solutions of different concentrations (20, 50, 100, and 200 mM). Briefly, a 2% (w/v) ALG solution was prepared by dissolving ALG powder in deionized water under magnetic stirring (500 rpm, room temperature) for 4 h until complete dissolution. The ALG solution was transferred into beakers separately, and an equal volume (5 mL) of CaCl_2_ solutions with the aforementioned concentrations was added dropwise to these beakers under continuous magnetic stirring to ensure homogeneous gelation through diffusion-mediated ionic crosslinking. The resulting hydrogels were allowed to stabilize for an additional 30 min at room temperature. Finally, the hydrogels were rinsed three times with deionized water to remove excess Ca^2+^ ions on their surfaces and equilibrated at room temperature for 2 h prior to further testing.

### 2.3 Swelling ratio measurement

The swelling behaviors of the alginate hydrogels crosslinked with different CaCl_2_ concentrations were evaluated in PBS (pH 7.4) at 37 °C. Briefly, pre-weighed hydrogel samples prepared at 50, 100, or 200 mM CaCl_2_ were immersed in PBS. At predetermined time points (0, 6, 12, 18, and 24 h), the samples were removed, gently blotted with a filter paper to remove excess surface liquid, and weighed immediately. The swelling ratio was calculated using the following formula:

$$\text{Swelling ratio }\left(\%\right)=\left(W_{t}-W_{0}\right)/W_{0}\times 100\%$$

where W_0_ is the initial weight of the hydrogel and W_t_ is the weight at each time point. The data are presented as mean ± SD.

### 2.4 Rheological measurements

Rheological time sweep measurements were performed using a rotational rheometer (TA Instruments, New Castle, DE, United States) having a parallel plate geometry (40 mm diameter). The measurements were conducted at 37 °C with a constant oscillatory strain of 1% and an angular frequency of 1 rad/s. The storage modulus (G′) and loss modulus (G″) were recorded as a function of time over a total duration of 5 min to monitor gelation kinetics.

### 2.5 Ca^2+^ release assay

The Ca^2+^ concentration in the hydrogel supernatants was quantified after a 72 h incubation period. Hydrogel specimens (n = 3 per group) were individually immersed in 5 mL PBS (pH 7.4) and incubated at 37 °C. After each predetermined time point (24, 48, and 72 h), the entire incubation medium was collected and replaced with an equal volume of fresh PBS. The collected supernatants were analyzed using an automated biochemical analyzer to determine the Ca^2+^ concentration. The cumulative Ca^2+^ release was calculated by summing the amount of Ca^2+^ released at each time point and normalizing it to the initial Ca^2+^ content in the hydrogel using the following equation:

$$\text{Cumulative release }\left(\%\right)=\left(\Sigma M_{t}/M_{0}\right)\times 100$$

where M_t_ represents the amount of Ca^2+^ released at each time point and M_0_ is the initial total Ca^2+^ content in the hydrogel.

### 2.6 Cell culture and proliferation assay

L929 fibroblasts were cultured in DMEM supplemented with 10% FBS and 1% penicillin–streptomycin (PS) at 37 °C in a humidified atmosphere containing 5% CO_2_. For proliferation assays, hydrogel extracts were prepared according to the ISO 10993–12 guidelines. Briefly, the hydrogels were soaked in serum-free DMEM for 24 h at 37 °C. L929 cells were seeded in 96-well plates at a density of 5 × 10^3^ cells/well and treated with 100 μL of hydrogel extracts for 1 day. Cell viability was assessed using a Cell Counting Kit-8 (CCK-8; MCE, United States) according to the manufacturer’s protocol. Absorbance was measured at 450 nm using a microplate reader (PerkinElmer, United States).

For EdU assays, cells were seeded at 1 × 10^4^ cells/well in 96-well plates and incubated with hydrogel extracts for 24 h. DNA synthesis was evaluated using an EdU incorporation assay (Beyotime, Shanghai, China) following the manufacturer’s instructions. Fluorescence images were captured using a fluorescence microscope (Olympus, United States), and the percentage of EdU-positive cells was quantified.

CCK-8 and EdU assays were performed with three independent biological replicates, each containing three technical replicates (n = 3).

### 2.7 Scratch wound migration assay

L929 cells were seeded in 6-well plates and cultured to ∼90% confluence. A uniform scratch was created with a 200-μL sterile pipette tip, and the detached cells were removed by washing with PBS. The cells were then incubated with hydrogel extracts in serum-free DMEM. Images of wounds were captured at 0 and 48 h using an inverted microscope (Olympus, United States). The migration rate was calculated as the percentage of wound closure using the ImageJ software.

Scratch wound migration assay was performed with three independent biological replicates, each containing three technical replicates (n = 3).

### 2.8 *In vivo* wound healing model

All animal experiments were conducted in accordance with the Guidelines for the Care and Use of Laboratory Animals and were approved by the Animal Ethics Committee of Nankai University (approval number: 2024-SYDWLL-000371). Male Sprague–Dawley (SD) rats (8 weeks old) were obtained from Beijing Vital River Laboratory Animal Technology Co., Ltd. SD rats (200–250 g) were anesthetized with 2% isoflurane, and a full-thickness excisional wound (diameter: 10 mm) was created on the dorsal skin. Further, rats were anesthetized with 2% isoflurane, and two full-thickness excisional wounds, 10 mm in diameter, were created symmetrically on the dorsal skin of each rat. The two wounds on the same animal received the same treatment. For *in vivo* wound healing analysis, wound closure was monitored on days 0, 7, 10, and 14. A total of six rats was used per group, with three rats sacrificed at each analyzed time point on days 7 and 14. The values from the two wounds on the same rat were averaged, and each rat was considered one biological replicate (n = 3 per time point). Wounds were randomly assigned to the following groups: (1) Gauze control and (2) ALG hydrogel crosslinked with 100 mM CaCl_2_. Dressings were applied immediately after wounding and replaced every 2 days. Wound closure was monitored by imaging, and the percentage of wound closure was calculated using the ImageJ software.

### 2.9 Histological analysis

Wound tissues were harvested on day 14, fixed in 4% paraformaldehyde, and embedded in paraffin. Sections (4 μm) were stained with H&E to assess re-epithelialization and granulation tissue formation and with Masson’s trichrome to evaluate collagen deposition. Ki-67 immunohistochemical staining was performed to assess cell proliferation. Images were captured using a light microscope.

### 2.10 RNA sequencing and bioinformatic analysis

Total RNA was extracted from the wound tissues collected on day 10 post-treatment using the TRIzol reagent (Invitrogen, Carlsbad, CA, United States) according to the manufacturer’s instructions. RNA integrity and quality were assessed using an Agilent 2100 Bioanalyzer (Agilent Technologies, Santa Clara, CA, United States). RNA libraries were constructed using the NEBNext Ultra RNA Library Prep Kit (New England Biolabs, Ipswich, MA, United States) and sequenced on an Illumina NovaSeq 6000 platform to generate 150 bp paired-end reads. Raw sequencing reads were filtered to remove adapter sequences, low-quality reads, and reads containing more than 10% ambiguous nucleotides (N) to obtain clean reads. Clean reads were aligned to the rat reference genome (Rnor_6.0) using HISAT2, and the gene expression levels were normalized as fragments per kilobase of transcript per million mapped reads (FPKM). Differentially expressed genes (DEGs) were identified using DESeq2, with thresholds of |log_2_ (fold change)| ≥ 1 and an adjusted p value of <0.05. The DEGs were further visualized based on the FPKM values. Functional annotation and pathway enrichment analyses, including Gene Ontology (GO) and Kyoto Encyclopedia of Genes and Genomes (KEGG), were performed using Omicsmart (https://www.omicsmart.com), with particular attention given to calcium-ion-mediated signaling pathways.

### 2.11 Statistical analysis

The quantitative data are presented as mean ± SD. For *in vivo* wound analysis, individual rats were considered biological replicates. When two wounds were created on the same rat, the average value of the two wounds was used for statistical analysis. Comparisons between two groups were performed using an unpaired Student’s t-test. Comparisons among multiple groups were performed using one-way ANOVA followed by Tukey’s *post hoc* test. A value of p < 0.05 was considered statistically significant.

## 3 Results and discussion

### 3.1 Effect of CaCl_2_ concentration on gel formation and mechanical properties

Alginate is a naturally occurring linear polysaccharide consisting of (1,4)-linked β-D-mannuronic acid (M) and α-L-guluronic acid (G) residues. According to the “egg-box” model, alginate polymers exhibit strong affinity for divalent cations, such as Ca^2+^, which preferentially bind to G-block regions, thereby facilitating ionic crosslinking between adjacent chains (Wang et al., 2023). Previous research has demonstrated that the Ca^2+^ concentration plays a critical role in modulating the physicochemical and mechanical properties of alginate hydrogels (Li et al., 2019). Building upon this strategy, we investigated the effects of varying CaCl_2_ concentrations on the gelation of a 2% ALG hydrogel.

**FIGURE 1:**
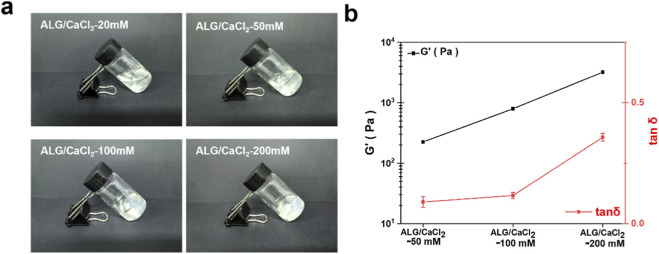
**(a)** Photographs of hydrogels crosslinked with 20, 50, 100, and 200 mM CaCl_2_. **(b)** Rheological characterization of ALG/CaCl_2_ hydrogels with varying CaCl_2_ concentrations.

As illustrated in Figure 1a, no gelation was observed at a CaCl_2_ concentration of 20 mM, whereas stable hydrogels were successfully formed at CaCl_2_ concentrations of 50, 100, and 200 mM. Additionally, rheological analysis was performed to characterize the gelation state and viscoelastic properties of the hydrogels (Supplementary Figure S1). At 20 mM CaCl_2_, the storage modulus (G′) remained consistently lower than the loss modulus (G″) throughout the measurement period, indicating predominantly viscous behavior and failure to form a stable gel network. By contrast, at 50 mM CaCl_2_, G′ became higher than G″ from the initial stage and increased over time, implying formation of a stable gel network. Moreover, as shown in (Figure 1b), the storage modulus increased with increasing CaCl_2_ concentration in the order of 50 mM < 100 mM < 200 mM. Notably, tanδ remained comparable between 50 and 100 mM, indicating a relatively stable viscoelastic balance within this concentration range. At 200 mM CaCl_2_, the hydrogel exhibited a substantially higher G′. In addition, tanδ slightly increased compared with that at lower concentrations, suggesting a change in the viscoelastic response at high crosslinking density.

To further characterize their properties, the swelling behaviors of the alginate hydrogels crosslinked with 50, 100, and 200 mM CaCl_2_ were evaluated in PBS. As shown in Supplementary Figure S2, all prepared hydrogels exhibited rapid swelling during the initial stage, followed by a more gradual increase over time. The swelling ratio decreased as the CaCl_2_ concentration increased. Specifically, the 50 mM group showed the highest swelling capacity, reaching approximately 45% at 6 h and 52% at 24 h, while the 100 mM and 200 mM groups reached approximately 32% and 25% at 24 h, respectively.

To evaluate the internal porous structure, the hydrogel prepared with 100 mM CaCl_2_ was analyzed via SEM (Supplementary Figure S3). A relatively homogeneous porous structure was observed internally. This may be related to more homogeneous diffusion of Ca^2+^ during gelation, which can reduce pronounced crosslinking gradients, possibly associated with gradual addition of CaCl_2_ under continuous stirring during fabrication.

### 3.2 Calcium ion release and its effect on L929 cell behavior

**FIGURE 2:**
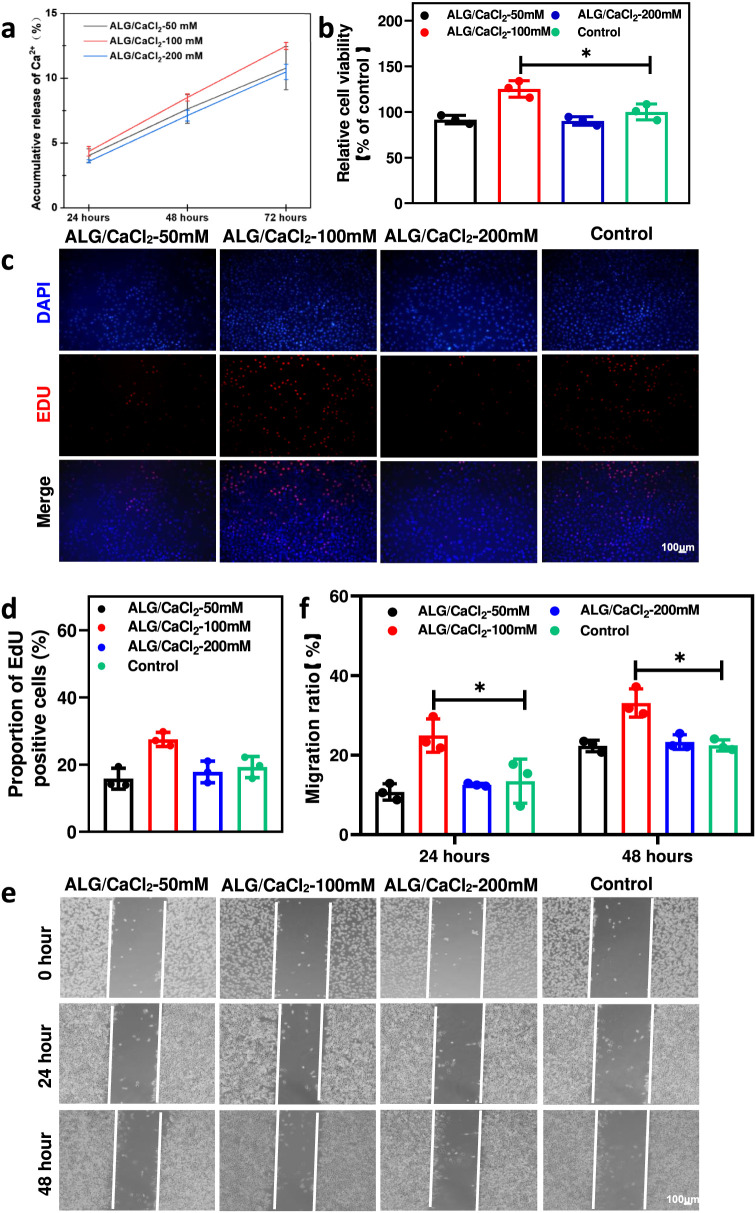
**(a)** Ca^2+^ release from alginate hydrogels after 24, 48 and 72 h immersion in PBS. Data are presented as mean ± SD (n = 3). **(b)** Cell viability of L929 fibroblasts cultured with hydrogel extracts assessed by CCK-8 assay after 48 h (n = 3). **(c)** Representative fluorescence images and quantification of EDU incorporation in L929 cells treated with hydrogel extracts for 48 h. EDU-positive cells are shown in red, nuclei were stained with DAPI (blue). Scale bar: 100 μm (n = 3). **(d)** Quantification of the proportion of EDU-positive cells in L929 fibroblasts after 48 h treatment with hydrogel extracts. Data are presented as mean ± SD (n = 3). **(e)** Representative images of scratch wound assays in L929 fibroblasts treated with hydrogel extracts at 0, 24, and 48 h. Scale bar: 100 μm (n = 3). **(f)** Quantification of fibroblast migration ratio at 24 and 48 h based on wound closure in the scratch assay. The wound gap distance between the two vertical lines was used to evaluate cell migration. Data are presented as mean ± SD (n = 3).

The release of calcium ions from the ALG/CaCl_2_ hydrogels was evaluated over a 72 h incubation period in PBS, as shown in Figure 2a. The hydrogels released Ca^2+^ in a time-dependent manner at 24, 48 and 72 h, indicating the gradual dissociation of ionic crosslinking within the hydrogel network. This sustained release profile reflects the diffusion-controlled behavior of Ca^2+^ from the alginate matrix (Lee and Mooney, 2012; Tavakoli et al., 2019).

Fibroblasts play a pivotal role in wound healing by producing extracellular matrix components, coordinating tissue repair, and modulating inflammation and scarring (Rahimi et al., 2022). To investigate the effect of the calcium ion concentration on the fibroblast behavior, we performed CCK-8 and EDU incorporation assays to evaluate cell proliferation (Navarro-Requena et al., 2018). L929 fibroblasts were cultured with the extracts of hydrogels crosslinked at varying CaCl_2_ concentrations. As shown in Figures 2b–d, the hydrogel crosslinked with 100 mM CaCl_2_ exhibited the most pronounced increase in fibroblast proliferation, as evidenced by increased cell viability and a higher proportion of EDU positive nuclei compared with the control and other hydrogel groups. Furthermore, scratch wound assays revealed that the hydrogel extracts promoted fibroblast migration, as shown in Figures 2e,f. At 24 h, the group treated with the hydrogel exhibited a significantly reduced wound gap compared with the control group, indicating enhanced migratory activity. Collectively, these findings suggest that the release of calcium ions within the first 48 h can positively influence fibroblast proliferation and migration, potentially contributing to accelerated wound healing *in vivo*.

Previous studies have similarly reported that ALG/CaCl_2_ dressings significantly increase fibroblast proliferation and activation *in vitro* (Hunt et al., 2013; Peltier et al., 2024). Additionally, fibroblasts encapsulated within ALG/CaCl_2_ gels maintain their viability and secrete angiogenic factors, including VEGF, over prolonged durations (Hunt et al., 2013). These findings are consistent with the hypothesis that extracellular calcium acts as a potent activator of fibroblasts. Nonetheless, effective activation is contingent upon an optimal extracellular Ca^2+^ concentration, as both deficiency and excessive levels of Ca^2+^ can adversely affect cellular function (Janssen et al., 2015; Feng et al., 2011). In addition, differences in the alginate composition, such as the mannuronic/guluronic (M/G) acid ratio, can influence Ca^2+^ chelation and release dynamics (Bennacef et al., 2023; Su et al., 2023), further affecting the biological responses of fibroblasts. Consequently, the enhanced proliferation observed in this system is more likely associated with a coordinated interplay between Ca^2+^ availability and the physicochemical properties of the alginate hydrogel, rather than being solely attributable to Ca^2+^ release.

To identify the most suitable hydrogel for subsequent studies, a systematic multiparameter comparison was conducted. As shown in Figure 1a, 20 mM CaCl_2_ failed to produce a stable hydrogel, while stable gels were obtained at 50, 100, and 200 mM. Rheological analysis revealed a concentration-dependent increase in the storage modulus (G′), with the 200 mM group showing the highest stiffness (Figure 1b; Supplementary Figure S1). At 200 mM CaCl_2_, the hydrogel exhibited a substantially higher G′. In addition, tanδ slightly increased compared with that at lower concentrations, suggesting a change in the viscoelastic response at high crosslinking density. The swelling capacity also decreased with increasing CaCl_2_ concentration (Supplementary Figure S2). Notably, the 100 mM formulation offered a favorable balance of adequate mechanical stability, moderate swelling, and suitable Ca^2+^ release (Figure 2a). *In vitro* assays further showed that 100 mM extracts promoted L929 fibroblast proliferation and migration more effectively than the 50 mM and 200 mM groups. Thus, the 100-mM CaCl_2_-crosslinked hydrogel was selected for *in vivo* evaluation because of its balanced properties and favorable effects on the fibroblast activity.

### 3.3 *In vivo* wound healing evaluation of ALG/CaCl_2_ hydrogel

**FIGURE 3:**
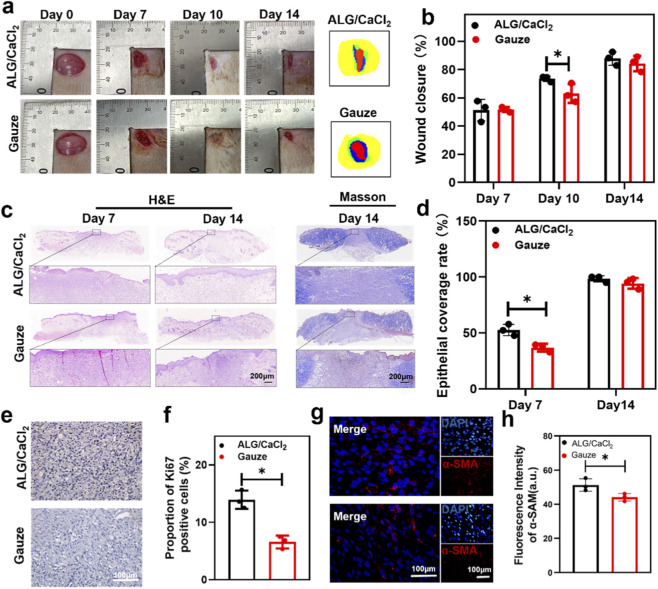
**(a)** Representative images of wounds on days 0, 7, 10, and 14. **(b)** Quantification of wound closure rates over time. **(c)** H&E and Masson’s trichrome staining of wound tissues. **(d)** Quantification of epithelial coverage. **(e)** Immunohistochemical staining of Ki67 showing proliferative cells at wound margins. **(f)** Quantification of Ki67-positive cells. **(g)** Immunofluorescence staining of α-SMA in tissue at the wound site. **(h)** Quantification of α-SMA fluorescence intensity.

A rat model with full-thickness skin defects was used to test whether the Ca^2+^-released ALG/CaCl_2_ hydrogels could accelerate wound healing (Luo et al., 2025). Considering its favorable mechanical properties and ability to promote the fibroblast activity, the 100-mM CaCl_2_-crosslinked alginate hydrogel (ALG/CaCl_2_-100 mM) was selected to evaluate the wound healing potential. Compared with the gauze-treated group, rats whose wounds were treated with the hydrogel exhibited accelerated closure and improved surface recovery throughout the observation period (Figures 3a,b). Histological analysis using H&E staining revealed a more complete epidermal coverage and a more organized dermal structure in the hydrogel-treated group than in the gauze-treated group (Figures 3c,d). Additionally, Masson’s trichrome staining revealed denser and better-aligned collagen fibers in the hydrogel group, indicating enhanced ECM remodeling and tissue maturation (Figure 3c). Thus, these results demonstrate that the ALG/CaCl_2_ hydrogels effectively promote wound healing by enhancing tissue regeneration and extracellular matrix organization.

To determine whether the observed acceleration of wound healing was mediated through activation of fibroblasts, we performed immunohistochemical staining for Ki67 to assess cell proliferation at the wound site (Bennacef et al., 2023; Su et al., 2023). Immunohistochemical staining for Ki67 indicated a higher density of proliferating cells at the wound edge in the hydrogel group, suggesting accelerated cell proliferation during the wound healing process (Figures 3e,f).

Together with the *in vitro* results showing enhanced fibroblast proliferation and migration, these findings suggest that ALG/CaCl_2_-100 mM promoted tissue repair through fibroblast-associated activity. To further evaluate the fibroblast-associated responses *in vivo*, α-SMA staining was performed on newly formed granulation tissues at the wound site at the same time point as that used in Ki67 analysis. Compared with the Gauze group, the ALG/CaCl_2_-100 mM group showed increased α-SMA-positive signals, suggesting enhanced myofibroblast activity during granulation tissue formation and wound healing (Figure 3g). Overall, the enhanced healing efficacy of the hydrogel crosslinked with 100 mM CaCl_2_ may be related to a more favorable balance between Ca^2+^ availability and hydrogel physicochemical properties. This CaCl_2_ concentration results in a higher crosslinking density, which contributes to enhanced mechanical stability (Wang et al., 2023; Kumar et al., 2023; Wang et al., 2024). The localized supply of Ca^2+^ likely stimulates the activity of fibroblasts, promoting collagen synthesis and cellular proliferation (Hunt et al., 2013; Peltier et al., 2024). This is evidenced by the increased expression of Ki67 and enhanced collagen deposition observed in wounds treated with the hydrogel.

### 3.4 Transcriptomic profiling reveals enrichment of the calcium signaling pathway

**FIGURE 4:**
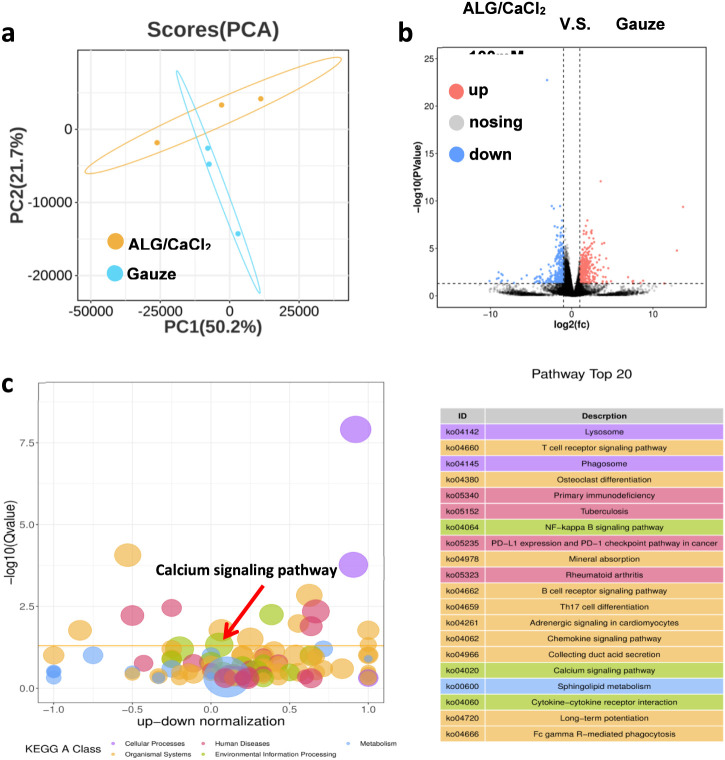
**(a)** Principal component analysis of transcriptomic data from hydrogel and gauze groups. **(b)** Volcano plot of differentially expressed genes between gauze and hydrogel groups. Red and blue points indicate significantly up- and downregulated genes, respectively (|log_2_FC | > 1, *p* < 0.05). **(c)** KEGG pathway enrichment analysis of differentially expressed genes.

To elucidate the molecular mechanisms responsible for the wound healing properties of the ALG/CaCl_2_-100 mM hydrogel, transcriptome sequencing was conducted on wound tissues from both the treated and control groups. Principal component analysis (PCA) revealed a distinct separation between the two groups, showing notable transcriptional modifications subsequent to hydrogel treatment (Figure 4a). Differential expression analysis revealed 316 genes as upregulated and 439 genes as downregulated (|log_2_FC| > 1, *p* < 0.05), thereby confirming significant transcriptional changes (Figure 4b). To better understand the biological relevance of these changes, representative differentially expressed genes related to fibroblast-associated functions were examined. Genes involved in extracellular matrix organization and remodeling, such as Col1a1, Adamts2 and Bgn, were upregulated, suggesting increased matrix deposition. Furthermore, genes associated with cell migration and adhesion, including, Ezr and Fscn1, were upregulated, consistent with the enhanced fibroblast migration observed *in vitro*. Additionally, changes were observed in several genes related to repair processes or cell cycle activity, including Cdc20 and Plk1, suggesting that the gene expression pattern may support tissue repair (Supplementary Table S1). Notably, classical proliferation markers, such as Mki67 and Pcna, did not show obvious changes, implying that the observed effects are more related to ECM remodeling and migratory activity than to a strong proliferative response.

KEGG enrichment analysis revealed significant enrichment of differentially expressed genes within the calcium signaling pathway (Figure 4c). This pathway is intricately linked to processes such as cell proliferation, migration, and extracellular matrix remodeling (Bonsignore et al., 2024). The observed enrichment implies that Ca^2+^ released from the crosslinked hydrogel not only contributes to the structural integrity of dressing but also serves as a bioactive mediator that initiates calcium-dependent signaling cascades. Taken together, these transcriptomic findings support the association of ALG/CaCl_2_ hydrogel treatment with calcium-signaling-related pathways and fibroblast-associated repair responses during wound healing.

This study has several limitations. The relatively small number of animals used at each time point may limit the statistical power of *in vivo* analysis. Future studies with larger sample sizes, *a priori* power calculations, and independent validation cohorts are required to further confirm the therapeutic efficacy of the ALG/CaCl_2_-100 mM hydrogels.

## 4 Conclusion

In this study, we evaluated the effect of Ca^2+^-crosslinked alginate hydrogels on wound healing. The ALG/CaCl_2_-100 mM hydrogel exhibited optimal mechanical properties and controlled Ca^2+^ release, which significantly promoted fibroblast proliferation and migration *in vitro*. Regarding *in vivo*, this hydrogel accelerated wound closure, enhanced epidermal regeneration, and improved collagen deposition and organization. Transcriptomic analysis further revealed activation of the calcium signaling pathway, confirming that the hydrogel affected healing through calcium-mediated stimulation of the fibroblast activity. Therefore, these findings demonstrate that ALG/CaCl_2_ hydrogels effectively facilitate wound repair by combining structural support with bioactive calcium release, highlighting their potential as versatile biomaterials for tissue regeneration applications.

## Data Availability

The datasets presented in this study can be found in online repositories. The names of the repository/repositories and accession number(s) can be found below: China National Center for Bioinformation/Beijing Institute of Genomics, Chinese Academy of Sciences (GSA: CRA032296) that are publicly accessible at https://ngdc.cncb.ac.cn/gsa.
