# Supplementary material for: Calcium ions released from alginate hydrogel promote wound healing by enhancing fibroblast activity
Source: Front Bioeng Biotechnol. 2026 Jun 19;14:1828848. doi: 10.3389/fbioe.2026.1828848 (PMC13327921; doi:10.3389/fbioe.2026.1828848)
Supplement: Supplementary file 1 [file DataSheet1.pdf]

Supporting Information

Calcium ions released from alginate hydrogel promotes wound healing by enhancing fibroblast activity

• Author names and affiliations.

Lei Zhang[a,b,c,1], Ying Luo[c,d,1,*], Shubo Liu[c,e], Huiting Deng[c,d], Yingtang Gao[c,d,f,*]

a School of Medicine, Nankai University, Tianjin 300071, China

b Department of Clinical Laboratory, Gansu Provincial Hospital, Lanzhou, Lanzhou 730000, China

c Tianjin Key Laboratory of Extracorporeal Life Support for Critical Diseases, Tianjin Institute of Hepatobiliary Disease, Nankai University Affiliated Third Center Hospital, Tianjin 300170, China

d Central Hospital, Tianjin University, Tianjin 300170, China

e The Third Central Clinical College of Tianjin Medical University, Tianjin, 300170, China

f Artificial Cell Engineering Technology Research Center, Tianjin, 300170, China

1 Lei Zhang and Ying Luo contributed equally to this work.

* Corresponding author.

Table of Contents

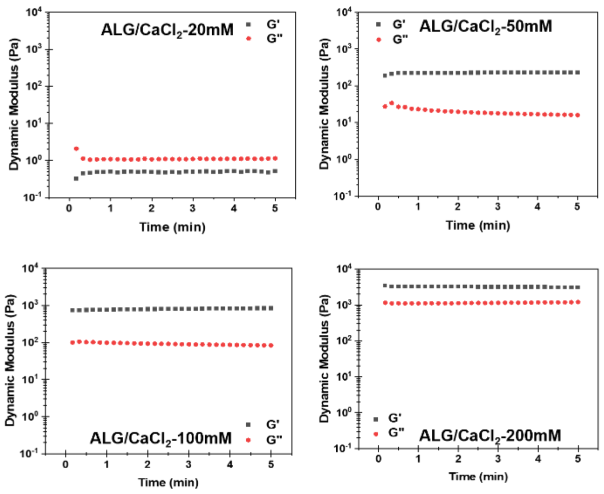

Figure S1. Rheological characterization of ALG/$CaCl_2$ hydrogels with varying $CaCl_2$ concentrations.

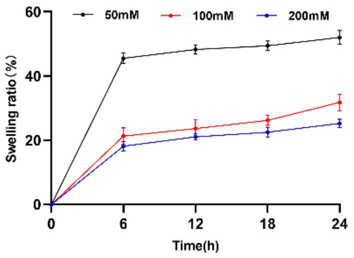

Figure S2. Time-dependent swelling behavior of ALG/$CaCl_2$ hydrogels with varying $CaCl_2$ concentrations.

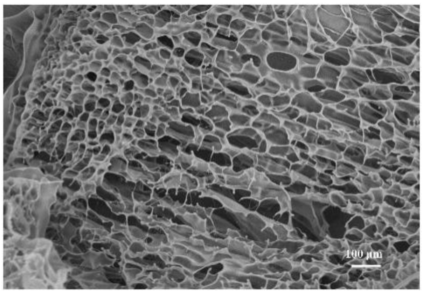

Figure S3. SEM images of ALG/$CaCl_2$-100mM hydrogels.

Table S1. Representative differentially expressed genes associated with extracellular matrix remodeling, migration/adhesion, and repair-supportive fibroblast responses in the hydrogel-treated group

Gene symbol	Category	F mean	G mean	log2FC (G/F)	P value	FDR	Interpretation
**Col1a1**	ECM remodeling	680.04	493.93	-0.462	0.0258	0.2940	Collagen synthesis
**Adamts2**	ECM remodeling	55.77	35.67	-0.649	0.0089	0.1673	Matrix remodeling
**Bgn**	ECM remodeling	494.43	380.77	-0.380	0.0094	0.1718	ECM assembly

**Ecm1**	ECM remodeling	40.52	20.13	-1.012	0.0167	0.2337	Matrix organization
**Mfap2**	ECM remodeling	24.04	14.72	-0.707	0.0368	0.3471	Microfibril remodeling
**Pcolce**	ECM remodeling	233.56	142.35	-0.714	0.0261	0.2953	Collagen maturation
**Col18a1**	ECM remodeling	11.14	6.82	-0.709	0.0404	0.3640	Structural support
**Ezr**	Migration/adhesion	448.43	326.41	-0.461	0.0257	0.2938	Adhesion/cytoskeleton
**Fscn1**	Migration/adhesion	18.68	11.31	-0.728	0.0258	0.2940	Migratory behavior
**Cxcr3**	Migration/adhesion	7.98	3.61	-1.140	0.0347	0.3397	Chemotactic signaling
**Cdc20**	Repair-supportive response	16.81	10.15	-0.721	0.0433	0.3770	Cell-cycle progression
**Cdc45**	Repair-supportive response	15.24	8.94	-0.770	0.0196	0.2521	DNA replication support
**Plk1**	Repair-supportive response	18.95	10.33	-0.874	0.0260	0.2951	Cell-cycle regulation
**Igfbp2**	Repair-supportive response	67.52	14.70	-2.200	0.0214	0.2648	Growth-supportive signaling
